# Genetic and demographic history define a conservation strategy for earth’s most endangered pinniped, the Mediterranean monk seal *Monachus monachus*

**DOI:** 10.1038/s41598-020-79712-1

**Published:** 2021-01-11

**Authors:** Alexandros A. Karamanlidis, Tomaž Skrbinšek, George Amato, Panagiotis Dendrinos, Stephen Gaughran, Panagiotis Kasapidis, Alexander Kopatz, Astrid Vik Stronen

**Affiliations:** 1 MOm/Hellenic Society for the Study and Protection of the Monk Seal, Solomou 18, 10682 Athens, Greece; 2 Department of Biology, Biotechnical Faculty, University of Ljubljana, Večna pot 111, 1000 Ljubljana, Slovenia; 3 Sackler Institute for Comparative Genomics, American Museum of Natural History, Central Park West at 79th Street, New York, NY 10024 USA; 4 Institute of Marine Biology, Biotechnology and Aquaculture, Hellenic Centre for Marine Research, Gournes Pediados, P.O. Box 2214, 71003 Heraklion, Crete, Greece; 5 Norwegian Institute for Nature Research (NINA), 7485 Trondheim, Norway; 6 Department of Biotechnology and Life Sciences, Insubria University, via J. H. Dunant 3, 21100 Varese, Italy

**Keywords:** Ecology, Genetics, Zoology, Environmental sciences

## Abstract

The Mediterranean monk seal *(Monachus monachus)* is a flagship species for marine conservation, but important aspects of its life history remain unknown. Concerns over imminent extinction motivated a nuclear DNA study of the species in its largest continuous subpopulation in the eastern Mediterranean Sea. Despite recent evidence of partial subpopulation recovery, we demonstrate that there is no reason for complacency, as the species still shares several traits that are characteristic of a critically endangered species: Mediterranean monk seals in the eastern Mediterranean survive in three isolated and genetically depauperate population clusters, with small effective population sizes and high levels of inbreeding. Our results indicated male philopatry over short distances, which is unexpected for a polygynous mammal. Such a pattern may be explained by the species’ unique breeding behavior, in which males defend aquatic territories near breeding sites, while females are often forced to search for new pupping areas. Immediate action is necessary to reverse the downward spiral of population decline, inbreeding accumulation and loss of genetic diversity. We propose concrete conservation measures for the Mediterranean monk seal focusing on reducing anthropogenic threats, increasing the population size and genetic diversity, and thus improving the long-term prospects of survival.

## Introduction

Anthropogenic pressures in the recent past have led to severe population declines^1^ and substantial fragmentation of wildlife populations^2^. As a result, small and fragmented wildlife populations are more vulnerable to genetic drift and inbreeding^3^, which, in turn may reduce their fitness and evolutionary potential, eventually driving species to extinction^4^. In such populations it is particularly important to thoroughly understand basic biological traits such as genetic diversity and structure and demographic history, as they are critical for planning any immediate or long-term conservation actions^3^. Such a science-driven approach has been used in the development and implementation of effective conservation measures, for example, in the genetic restoration of the Florida panther (*Puma concolor coryi*)^5^; in contrast, the lack of information about demographic history and genetic diversity has hampered the development of conservation measures in the vicuña (*Vicugna vicugna*)^6^.

The Mediterranean monk seal (*Monachus monachus*) is arguably the most endangered pinniped in the world^7^ and a flagship species for marine conservation. Following centuries of human persecution and habitat loss, the species has been extirpated from most of its historical range and survives in three small, isolated subpopulations: two in the northeastern Atlantic (i.e., Cabo Blanco and archipelago of Madeira) and one in the eastern Mediterranean Sea^8^. The latter subpopulation currently comprises more than 90% of the species’ area of occupancy, is estimated to number fewer than 300 mature individuals^9^, and has been a focal point of systematic conservation efforts since the early 1990s^10^.

Despite recent encouraging signs of partial population recovery^10^, the survival of the Mediterranean monk seal in the eastern Mediterranean is far from secure. Mediterranean monk seals are notoriously elusive and challenging to study, which has made the wide-scale application of traditional monitoring techniques, such as photo-identification and telemetry, logistically challenging. Our limited understanding of the species’ genetic and demographic history and status of populations has been one of the main impediments in designing and implementing effective conservation measures^8^.

Molecular genetics has repeatedly been shown to be an invaluable tool in studying and ultimately protecting elusive and/or endangered species^11^. A previous nuclear DNA (nDNA) study of the Cabo Blanco and the eastern Mediterranean monk seal subpopulations, using 24 microsatellite loci, recorded low intrapopulation diversity, substantial genetic differentiation between subpopulations and no obvious population structure within each subpopulation^12^. However, the findings for the eastern Mediterranean subpopulation should be interpreted with caution, due to the small sample size (n = 12) and the marked differences in demographics, ecology and behavior between these two subpopulations^8^. A more recent genetic study of the Mediterranean monk seal in the eastern Mediterranean using mitochondrial DNA (mtDNA) markers, has suggested that the species is one of the most genetically depauperate mammals on earth^13^.

**Figure 1:**
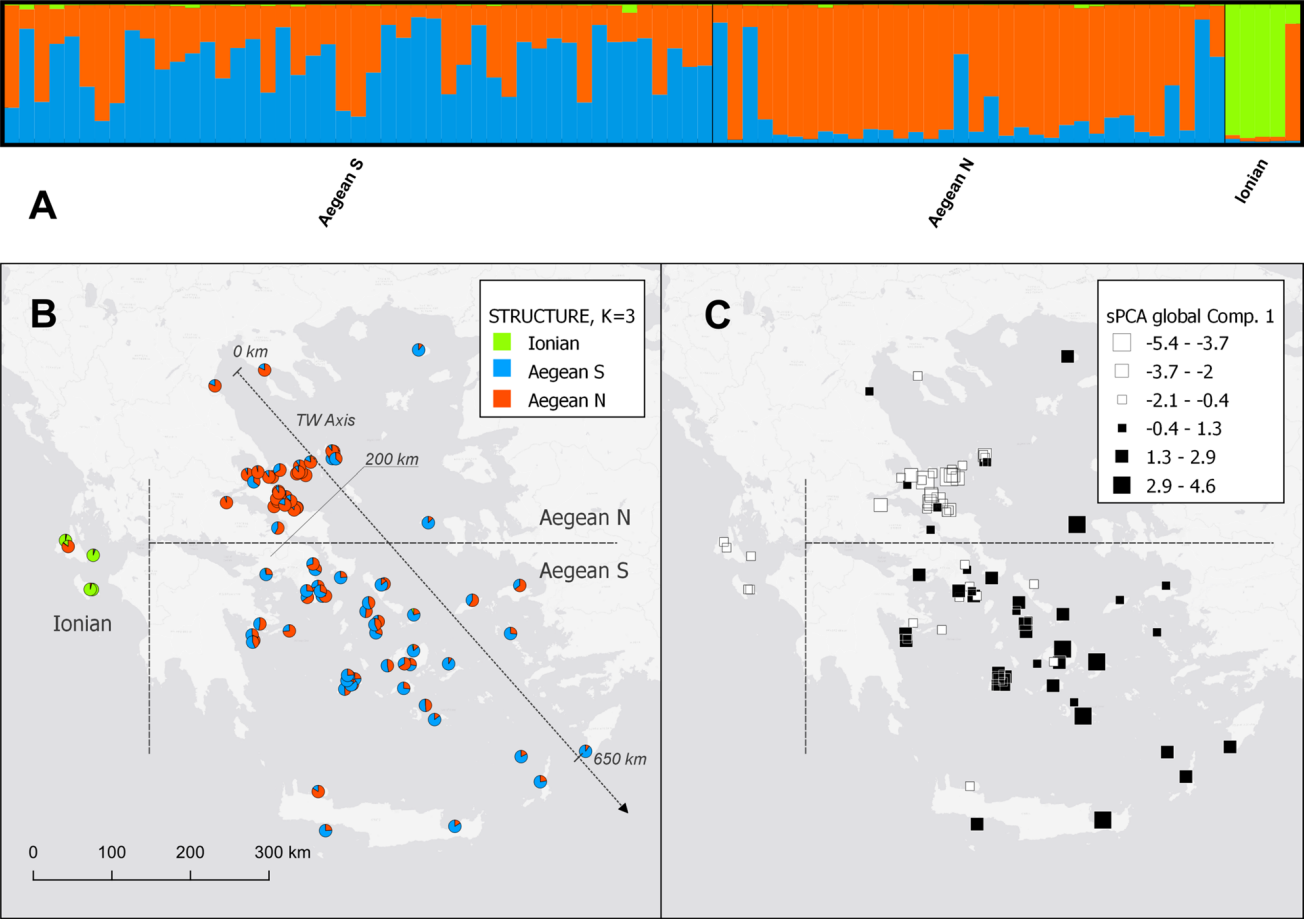
Population structure results for Mediterranean monk seals in Greece with 26 microsatellite markers and *K* = 3 population clusters. (**A**) Plot of estimated *Monachus monachus* population assignment coefficients from the STRUCTURE model, where sampling locations were used as a location prior (LOCPRIOR). Each vertical bar represents assignment coefficients for a single individual; (**B**) Map of Greece with the direction and distances of the travelling window (TW) axis for the Hardy–Weinberg Dynamic Subsampling analysis and the individual results of the STRUCTURE analysis. (**C**) Map of Greece with the results of the sPCA analysis, global Component 1 (Fig. 1b,c have been created using QGIS (Version 3.16.0. https://qgis.org/).

The dire genetic situation of the Mediterranean monk seal, our limited understanding of its life history and the low number of surviving animals call for immediate scientific attention, and for effective, concrete conservation actions. In this study we used nDNA microsatellite markers, which have a higher resolution and reflect recent population processes^14^, to assess the conservation status of the species in the eastern Mediterranean Sea from a genetic perspective. We analyzed contemporary genetic samples from Mediterranean monk seals in Greece (Fig. 1B), covering most of the eastern Mediterranean monk seal subpopulation, the largest subpopulation still in existence^9^ to address questions that have been identified as essential in the global management of the species^15^: (1) What is the current population structure of Mediterranean monk seals in Greece and how is gene flow maintained? (2) What are the levels of genetic diversity of the monk seal subpopulation in Greece and how is this diversity distributed geographically? (3) What are the levels of individual inbreeding and what is the effective population size (*N*_*e*_) of the Mediterranean monk seal clusters in Greece? Based on our current understanding of the recent demographic history of the species and considering the central position of Greece within the eastern Mediterranean monk seal population, we expected limited population structure and gene flow that is mediated by male dispersal, medium to low levels of genetic diversity, and medium levels of inbreeding and small effective population sizes.

Our study system provides a unique opportunity to demonstrate how hard-to-obtain information on basic genetic and demographic parameters of elusive and endangered species is indispensable in developing science-based conservation strategies for their recovery. We use our findings to develop a science-based conservation strategy with concrete management recommendations that will increase this charismatic flagship species’ chances of survival.

## Results

### Population structure and dispersal

STRUCTURE analyses indicated that the most likely number of genetic clusters was *K* = 3 (Figs. S1–S4). These three clusters represented individuals sampled in the Ionian, the northern Aegean, and the southern Aegean Sea, respectively (Figs. 1A–C, S2, S4). Four out of the five individuals sampled in the Ionian Sea were assigned with high probability (q_i_ ≥ 0.94) to the Ionian cluster. One individual in the Ionian exhibited substantial proportion of ancestry attributed to the northern Aegean cluster (Fig. 1B). Of the 45 females and 39 males sampled in the study, five (11.1%) females and two (5.1%) males were classified as migrants (See Supplementary Information for details on the results of the STRUCTURE analysis).

sPCA results indicated global structuring (*P* < 0.001), with two seemingly interpretable global components, and no local structuring (*P* = 0.719) (Fig. S5). Global component 1 (eig = 2.399) indicated structuring between Mediterranean monk seals in the northern and southern Aegean Sea (Fig. 1C), while global component 2 (eig = 1.863) separated Mediterranean monk seals in the Ionian Sea as a distinct population cluster (Figs. S6, S7).

**Figure 2:**
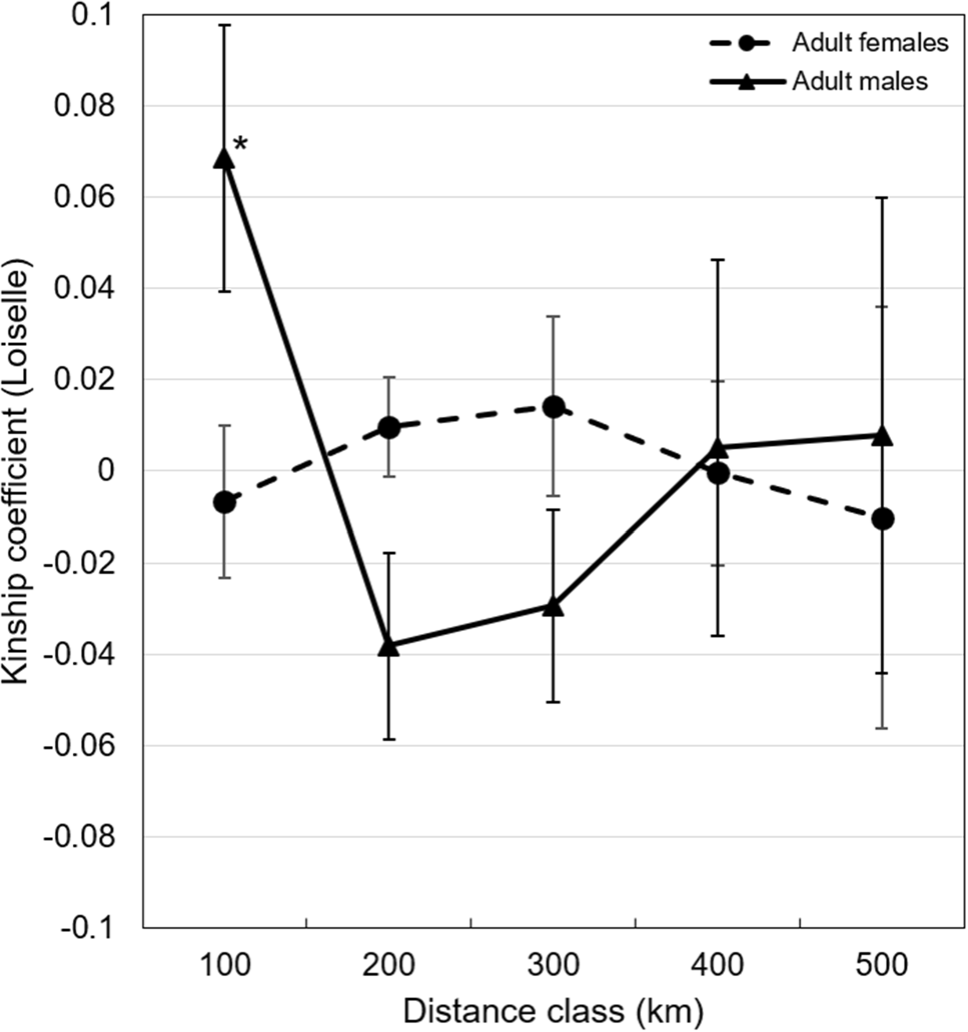
Results of the isolation-by-distance analysis among adult female and male Mediterranean monk seals in Greece. Distance classes that differed significantly (*P* < 0.05) from the mean kinship of the population are marked with an asterisk (*).

Adult male seals displayed a distinctive Isolation by Distance (IBD) pattern until a distance of 100 km (*P* = 0.0172). Within this distance class, pairwise comparisons among individuals showed significantly higher values than the population’s mean kinship value (Fig. 2). At larger distances, kinship did not differ among the distance classes. For adult females, kinship values between all distance classes did not differ significantly.

### Genetic diversity and HWDS analysis

Heterozygosity and allelic diversity were generally low, and no statistically significant differences between genetic clusters were found (Table 1). Results for the Ionian Sea were based on only five genotypes and were therefore difficult to interpret.

**Table 1:** Genetic diversity indices and estimates of effective population size for Mediterranean monk seal population clusters in Greece, summarized for 26 microsatellite loci. *N*: number of individuals; *He*: expected heterozygosity; *Ho*: observed heterozygosity; *A*: number of alleles. *SE*: Standard Error; *N HWE*: the number of loci statistically significantly departing from the Hardy Weinberg Equilibrium (HWE) with *P* < 0.05; *Ne*: effective population size, with 95% confidence interval in the parentheses. Effective population size was not calculated for the Ionian due to the small sample size.

Pop	*N*	*He*	SE *He*	*Ho*	SE *Ho*	*A*	SE *A*	*N HWE*	*Ne*
Northern Aegean	34	0.370	0.040	0.368	0.043	2.667	0.216	1	28.5 (19.3–47.3)
Southern Aegean	47	0.361	0.043	0.328	0.041	2.833	0.254	2	66.8 (44.0–120.9)
Aegean all	81	0.372	0.041	0.345	0.040	3.033	0.277	5	50.4 (39.8–65.7)
Ionian	5	0.318	0.043	0.371	0.063	2.033	0.155	0	N/A

**Figure 3:**
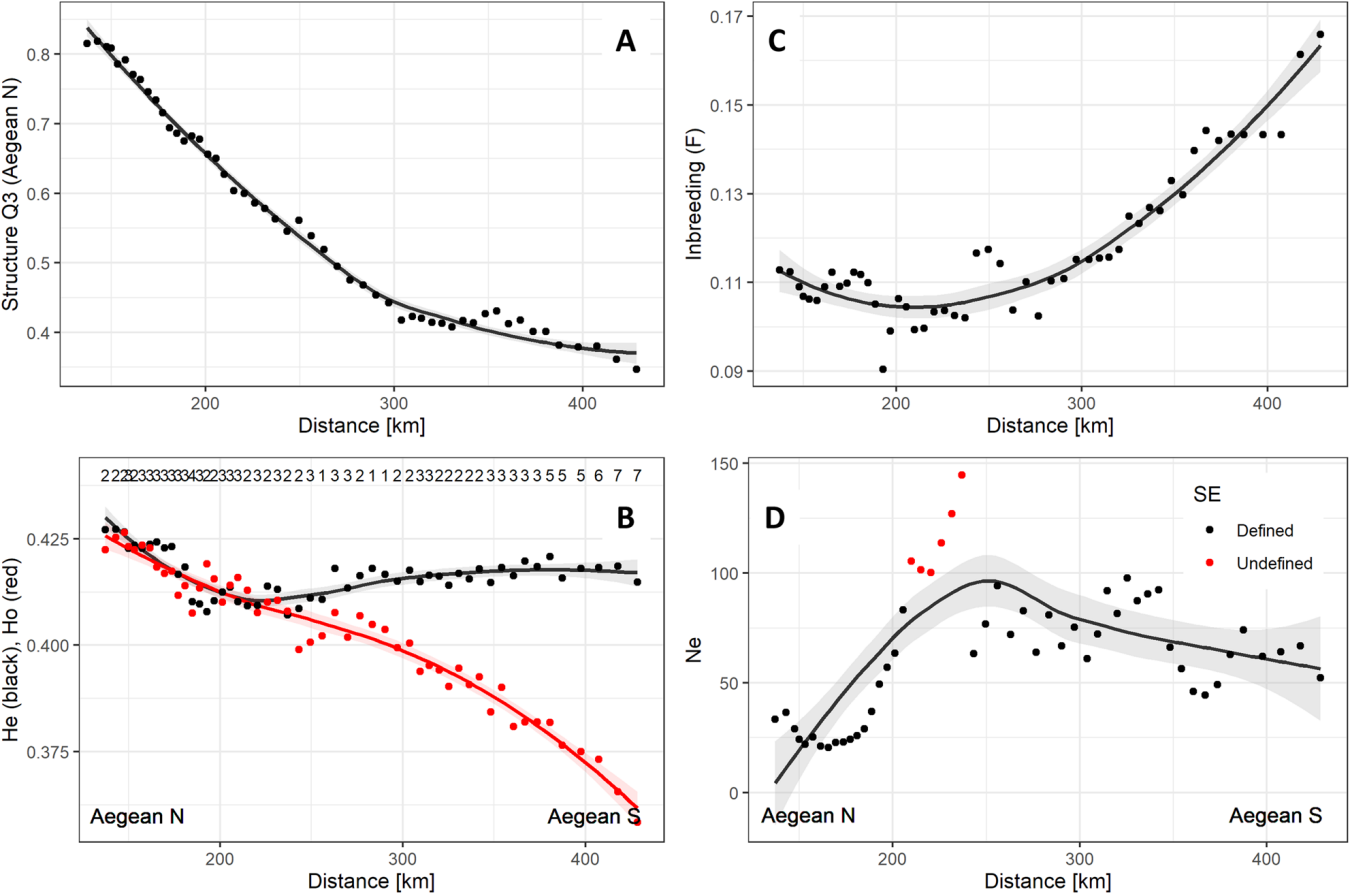
Hardy–Weinberg Dynamic Subsampling analysis results for Mediterranean monk seals in the Aegean Sea. “Distance” is the distance along the travelling window (TW) axis (see Fig. 1C). (**A**) Structure assignment probability to the Aegean North (Q3); (**B**) Expected (*He*; black) and observed (*Ho*; red) heterozygosity estimates for Mediterranean monk seals in the Aegean Sea. The numbers on the top indicate the number of loci out of Hardy Weinberg Equilibrium at *P* < 0.05. (**C**) Individual inbreeding estimates (TrioML estimate) along the TW axis; (**D**) Effective population size estimates along the TW axis. Red dots indicate estimates where Standard Error (SE) could not be reliably estimated.

The northern Aegean population cluster (Fig. 1B) was clearly defined, with most individuals in the area assigned to it with a high probability (Fig. 3A). The southern Aegean population cluster was less distinct, with many admixed individuals (Fig. 1A-B).

Expected (*He*) and observed (*Ho*) heterozygosity estimates were similar in the North Aegean, but started diverging approximately 200 km to the South along the TW axis, indicating deviations from Hardy–Weinberg equilibrium (HWE), with seven (23%) of the markers showing statistically significant departure from HWE (Fig. 3B).

### Individual inbreeding

COANCESTRY simulations indicated that the TrioML provided the lowest bias and the highest precision. The estimates seemed unbiased, but the precision of individual estimates was relatively low (Fig. S8), indicating that the results were useful for evaluation of average inbreeding for many animals, but less useful for evaluation of inbreeding at the individual level. The HWDS analysis indicated that inbreeding increased from northwest (median F = 0.03, IQR = 0.12) to southeast (median F = 0.11, IQR = 0.19) (Fig. 3C). Individuals in the northern Aegean were in general less inbred than the individuals sampled in the southern Aegean (Fig. S9) (See Supplementary Information for details on the results of the individual inbreeding analysis).

### Effective population size

The *N*_*e*_ estimate of the southern Aegean population cluster was higher than that of the Northern Aegean (66.8 vs 28.5, Table 1). The *N*_*e*_ estimates for the northern Aegean provided narrow confidence intervals, much narrower than those for the southern Aegean. HWDS analysis indicated low *N*_*e*_ estimates in the North, which increased rapidly around 200 km along the TW axis (Fig. 3D).

## Discussion

We applied genetic tools to assess the conservation status of the largest remaining subpopulation of Mediterranean monk seals, the subpopulation in the eastern Mediterranean Sea. We found a clear division of Mediterranean monk seals in Greece into three genetic clusters: one cluster in the Ionian Sea and two clusters in the Aegean Sea.

All individuals sampled in the Ionian had the distinct MM03 mtDNA haplotype that has not been detected in the Aegean^13^, and all individuals except the one assigned to the Aegean cluster using nuclear DNA had 1–2 microsatellite alleles only found in the Ionian Sea. This indicates that the Ionian cluster carries a portion of the Mediterranean monk seals’ evolutionary heritage that is not found in the Aegean and should therefore be considered an important unit for conservation.

In the Aegean Sea, the higher number of samples allowed us to explore the recent demographic and genetic history of the Mediterranean monk seal in the region in more detail. The northern cluster had low inbreeding, low *N*_*e*_ and little deviation from HWE, and it did not seem to have been receiving much gene flow from the other two clusters. Due to anthropogenic disturbances (e.g., habitat fragmentation, deliberate killing) the animals in this cluster were most likely isolated from other Mediterranean monk seals, with genetic drift rapidly forging a unique genetic signature. Active conservation in the northern Aegean started in the early 1990s^10^ and likely facilitated population recovery in the area.

In the southern Aegean the situation appeared different and more complicated. While there are continuous historic records of Mediterranean monk seals in the region, there is ample evidence of increased human persecution of the species in the 1900s^8^, which likely led to a sharp population decline. This in turn resulted in reduced gene exchange with other Mediterranean monk seal populations, causing a high degree of genetic separation. The southern Aegean population started inbreeding more rapidly than the northern cluster, with genetic drift ultimately causing the allelic frequencies to change enough to detect it as a unique genetic cluster. Inbreeding and genetic difference to the northern cluster decreased towards the North, indicating that admixture between the two genetic clusters is relatively recent. This also had a clear effect on *N*_*e*_, which was higher in the “admixing zone” (Fig. 3D, distance > 200 km). Another possible explanation for the observed genetic structuring could be gene flow from a (possibly) so far unidentified, genetically different Mediterranean monk seal population to the east. However, if this were the case, one would expect to detect increased genetic diversity in the “admixing zone” with the north, which we did not see. Expected heterozygosity (*He*) did not increase during the process of admixing of the clusters, indicating that the southern Aegean population is not contributing much unique genetic diversity. The pattern of deviations from HWE in the South Aegean with increasingly lower *Ho* compared to *He* as we move towards south (Fig. 3B), paired with a gradient in STRUCTURE assignment probabilities (Fig. 3A) and increasing inbreeding (Fig. 3C) indicate a possible signal of assortative mating in the far south, a genetic signature of past genetic isolation and bottleneck, which dissolves as we move north.

As with Mediterranean monk seals in the Atlantic^16^, the genetic diversity of Mediterranean monk seals in Greece was extremely low, with each of the three population clusters having an average of fewer than three alleles across the 26 loci investigated. Moreover, microsatellites are often biased toward highly polymorphic loci with rapid mutation rates^17^, and can give relatively high diversity values that may not be representative when considering the overall genome of the species. In our study we also excluded the monomorphic loci to have a consistent dataset in all analyses, which probably biased the diversity estimates somewhat higher. In general, low genetic diversity is expected to have detrimental effects on individual fitness and adaptability and, consequently, on population persistence and survival^18^. Across taxa, several populations of wide-ranging species display low genome-wide diversity across genetic markers, including ungulates^19^, felids^20^ and canids^21^. Immigrants were detected in all three clusters, further supporting the finding that individuals are admixing across clusters. Encouragingly, genetic diversity and fitness have been found to improve even with a low number of migrants^21,22^, and also in cases of gene flow between populations that exhibit both, low levels of genetic diversity and high levels of inbreeding^23^. This fact underlines the importance of increasing connectivity among clusters in order to increase genetic diversity, which is especially challenging for small and fluctuating populations that often require a high number of migrants for long-term survival^24,25^.

*N*_*e*_ is one of the most important parameters for conservation as it describes the sensitivity of a population to genetic stochasticity, as well as its evolutionary potential^26^. This parameter, however, is often difficult to assess from real-world data and should be interpreted with caution (See Supplementary Information for a detailed discussion). The *N*_*e*_ for the northern Aegean cluster does not meet the *N*_*e*_ > 50 criterion that is thought to allow a population to avoid inbreeding and it is still far below the rule-of-the-thumb minimum threshold of *N*_*e*_ > 500 that has been suggested to maintain genetic diversity^18^. Even the admixing of population clusters, which increased overall *N*_*e*_, merely made the entire Aegean population marginally viable in the sense that it might be able to avoid inbreeding. However, it has been argued that for endangered species the target effective size for a conservation program should be in the range of 1000–5000^27^. In this regard, our *N*_*e*_ for the entire Aegean monk seal population is more than an order of magnitude lower than the minimum effective population size conservatively recommended for the management of endangered species.

Adult males appeared to be more related until a distance of 100 km, while adult females displayed no correlation between kinship and geographical distance. Thus, IBD is likely to have influenced the genetic structure among males at shorter distances. Male dispersal and female philopatry appears to be the norm in polygynous mammals^28^, such as the Mediterranean monk seal^8^. However, assessing dispersal in marine mammals is notoriously challenging. To this date, findings of sex-biased dispersal in pinnipeds has remained inconclusive. For example, female dispersal has been described in Southern elephant seals (*Mirounga leonina*)^29^ and long distance dispersal has been reported for both sexes^30,31^. The pattern indicated by our results may be explained by the unique breeding behavior of the Mediterranean monk seal, where females need to find new pupping areas due to limited space in existing ones^32^ and males tend to defend aquatic territories around pupping sites^33^. Most likely as a result of this, female Mediterranean monk seals have often been directly observed to disperse in the eastern Mediterranean^8^. Consequently, the fact that no dispersal of individuals from the Ionian to the Aegean has been documented so far (results of this study and^13^), indicates that the number of Mediterranean monk seals in the Ionian Sea is comparably low and that there is still suitable pupping habitat available in the region. Kinship coefficients in our study were moderately low, but similar to those described for e.g., the Saimaa ringed seal (*Phoca hispida saimensis*)^34^. While long-distance dispersal in adult female seals is likely^29^ and has been recorded in the species^8^, general conclusions on sex-biased dispersal in the Mediterranean monk seal cannot be drawn. We advocate for a targeted study combining movement (e.g., telemetry) and genetic data to comprehensively assess the species’ dispersal behavior.

Studying Mediterranean monk seals in the wild is demanding: animals are rare, spread over a vast geographical scale (i.e., Greece has > 16,000 km of coastline), and only a small fraction of dead seals is typically recovered. This makes genetic sampling difficult, and sample sizes that are regularly obtained in many other species of conservation concern are virtually impossible to obtain in such an elusive species. On the other hand, due to the extremely small population size of this species, our current sampling represents a significant proportion of the monk seal population in the eastern Mediterranean, which has allowed a comprehensive and representative genetic assessment of the species in the region. The data that we have managed to obtain should be used to develop effective conservation policies that may as well prove to be crucial for ensuring the species’ survival.

Despite recent encouraging signs of partial population recovery^10^, our genetic assessment of Mediterranean monk seals in Greece paints an alarming picture of the species’ genetic status, raising questions on how close the eastern Mediterranean monk seal subpopulation may actually have been to extinction, and highlighting the urgent need to step-up the efforts to protect it. While Greece hosts one of the largest Mediterranean monk seal subpopulations worldwide, even here the species survives in a fragmented population with low genetic diversity, small effective population size, and high levels of inbreeding. Considering that the main threats to the Mediterranean monk seal are anthropogenic^8^, we believe that human impacts on the species’ behavior (e.g., pupping in marine caves^7^) have played a significant role in shaping this picture. On the other hand, gene flow among previously isolated clusters is being re-established, giving ground for cautious optimism. We believe that the dispersal of Mediterranean monk seals from the northern Aegean nucleus towards the south is direct evidence of the effectiveness of the systematic conservation actions that have been in place in the area since the early 1990s, which include protecting critical pupping habitat^32^ and regulating human activity^35^.

Based on our findings, we believe that reversing the downward spiral of population decline, inbreeding accumulation, and loss of genetic diversity while creating favorable conditions for a population increase must be priorities for conservation. We recommend that conservation actions for the Mediterranean monk seal in Greece should focus primarily on reducing anthropogenic threats, by: (a) Identifying and effectively protecting the most important pupping sites in the country, thus improving habitat quality and promoting effective dispersal and gene flow. (b) Increasing monitoring and protection of Mediterranean monk seals in the Ionian Sea to better understand their conservation status, but most importantly, to preserve their unique genetic diversity and encourage gene flow from the Ionian to the Aegean Sea. In a species so genetically impoverished as the Mediterranean monk seal, it is invaluable to maintain any genetic diversity still in existence. If population demographics permit, gene flow could also be artificially enhanced through the release of rehabilitated individuals found in the Ionian into the Aegean Sea and vice versa. (c) Reducing anthropogenic mortality (i.e., deliberate killing and accidental entanglement in fishing gear^8^). This could be supported by the establishment of a compensation system to alleviate financial losses from damages to fishing gear by Mediterranean monk seals^36^. (d) Since monitoring is essential for any conservation effort and considering the wealth of information that genetic monitoring of Mediterranean monk seals in Greece has provided, we believe it is essential that it is continued within the framework of the Hellenic Monk Seal Register^37^, i.e., a project dedicated to genetically monitoring Mediterranean monk seals in Greece. These recommendations make up the core of the newly-formulated Conservation Action Plan for the Mediterranean monk seal in Greece^10^, and should play an essential role in future conservation actions for the species in Greek waters.

But, at the end of the day, is it really worthwhile investing in the conservation of a species that is already in such a precarious genetic state, and what are the chances of succeeding? The Mediterranean monk seal is just one species on the long list of pinnipeds whose genetic state has been severely affected by humans^38^. Although some evolutionary heritage in the Mediterranean monk seal has certainly been irreplaceably lost (see also^15,39^), a rapid population increase would reduce genetic drift, loss of variability and inbreeding accumulation, improving the prospects for long-term survival^40^. Following the implementation of strict conservation measures, a close relative of the Mediterranean monk seal, the northern elephant seal (*Mirounga angustirostris*), was able to recover from a similar bottleneck despite severe loss of genetic diversity^41^, giving also hope for the future recovery of the Mediterranean monk seal. Our findings on the movement of Mediterranean monk seals out of the Northern Aegean show that the recipe of habitat protection and reduced anthropogenic mortality is a successful one. It remains upon humans to see this bigger picture and ensure that these effective conservation measures are continued and expanded to safeguard the future of the species.

## Methods

### Sample collection and molecular analysis

Eighty-six tissue samples were collected during the necropsies of Mediterranean monk seals found throughout Greece (1994–2016) and stored in 95% ethanol or DMSO at − 20 °C. Genomic DNA was extracted using the Qiagen DNeasy^®^ Blood & Tissue extraction kit, following the manufacturer’s protocol. Tissue samples were genotyped using 30 microsatellite loci^42–48^ (Table S1). Samples were run on an ABI3730 and electropherograms were analyzed in GeneMapper (version 5.0, https://www.thermofisher.com/order/catalog/product/4370784#/4370784). Four loci were found to be monomorphic in our population and excluded from the downstream analysis (See Supplementary Information for details on the molecular genetic analysis).

### Population structure and dispersal

Genetic structure was explored using two approaches. First, to explore population structure and determine individual assignment to clusters, a Bayesian Markov Chain Monte Carlo (MCMC) clustering method was employed using the software STRUCTURE ver. 2.3.4^49^. STRUCTURE was run five times for the optimal K value, incorporating 90% probability intervals for cluster membership (q_i_). The results with the highest likelihood L(K) and lowest variance were chosen. To obtain meaningful population units where only individuals with a high degree of membership were considered, individuals were assigned to a cluster k when q_i_ ≥ 0.8 to that cluster and probability intervals excluded membership in alternative clusters (See Supplementary Information for details on the STRUCTURE analysis).

To further investigate and visualize population structure, a spatial Principal Component Analysis (sPCA)^50^ was performed, following the procedure described by^54^. We modelled spatial connectivity using the Delaunay triangulation^51^ and used the sPCA scree plot to visually determine the number of components to be interpreted, and Monte Carlo tests with 10,000 permutations to test for the existence of global and local spatial structure^50^. We ran the analysis in the R statistical environment^52^ using the *adegenet* package^53^.

Finally, to understand the drivers affecting population structure, and considering that in widely-dispersing species isolation-by-distance (IBD) likely affects population composition^54^, we tested for IBD of adult females and males using the software SPAGEDI 1.5^55^. The program combines pairwise comparisons of estimated kinship coefficients^56^ and spatial distance between individual sampling locations, based on longitude and latitude coordinates. We obtained significant departures from the mean kinship coefficient of each distance class by 20,000 permutations and standard errors were estimated by jackknifing over loci^55^.

### Genetic diversity and HWDS analysis

Genetic diversity parameters for each Mediterranean monk seal population cluster in Greece were calculated using the R package *adegenet*^53^. Nuclear DNA diversity was measured as the number of alleles per locus (*A*), the observed heterozygosity (*H*_*o*_) and Nei’s unbiased expected heterozygosity (*H*_*e*_)^57^, and deviations from Hardy Weinberg Equilibrium (HWE) were tested using the likelihood ratio-based exact test described by^58^, as implemented in the R package HWXtest^59^. When a full enumeration was not feasible, the Monte Carlo method was used, with the cut-off value set to 10^9^ tables.

To understand the distribution of genetic diversity and the main drivers shaping it (i.e., explore the size and direction of local deviations from Hardy–Weinberg proportions), a Hardy–Weinberg Dynamic Subsampling analysis (HWDS) was performed^60^. The geographic northwest-southeast axis (Fig. 1B) was used as the travelling window path and each “window” was defined as 30 geographically consecutive genotypes along this path. For each window the genetic diversity parameters, average assignment probability to STRUCTURE clusters, average individual inbreeding and effective population size (*N*_*e*_) were calculated in the R statistical environment^52^, using functions from the *adegenet* package (See Supplementary Information for details on the HDWS analysis).

### Individual inbreeding

Individual inbreeding was estimated using the methods implemented in the software COANCESTRY (Version V1.0.1.9, https://www.zsl.org/science/software/coancestry)^61^. Initially, simulations were used to determine the power of the marker set and select the most appropriate method. The observed allelic frequencies at the actual markers analyzed in this study were used to parametrize simulations. We simulated genotypes with different levels of inbreeding between 0.05 and 1, in steps of 0.05, 100 individuals in each category. Inbreeding in simulated data was estimated using four of the methods implemented in the software COANCESTRY (Ritland, LynchRd, TrioML, DyadML) and the estimates were compared with the simulated “true” values. The best-performing method was used for calculating the final empirical individual-level inbreeding estimates.

### Effective population size (N_*e*_)

The effective population size of Mediterranean monk seal population clusters was estimated using the unbiased linkage disequilibrium (LDNe) estimator^62^. In small populations the method is reasonably precise and unbiased already at sample sizes of 25 individuals^62,63^. We applied the method using the NeEstimator program^64,65^, following the recommendations of Waples & Do^63^, and excluded rare alleles with frequencies below 0.02.

## Supplementary information

Supplementary Information 1.

Supplementary Information 2.

## Data Availability

All genetic data applied in this study have been made available in the Supplementary dataset.
