# Supplementary material for: Genetic and demographic history define a conservation strategy for earth’s most endangered pinniped, the Mediterranean monk seal Monachus monachus
Source: Sci Rep. 2021 Jan 11;11:373. doi: 10.1038/s41598-020-79712-1 (PMC7801404; doi:10.1038/s41598-020-79712-1)
Supplement: Supplementary file 2 — Supplementary Information 2. [file 41598_2020_79712_MOESM2_ESM.docx]

**Supplementary information**

**TITLE PAGE**

**Genetic and demographic history define a conservation strategy for Earth’s most endangered Pinniped, the Mediterranean monk seal *Monachus monachus***

Alexandros A. Karamanlidis^1,7,*^, Tomaž Skrbinšek^2,7^, George Amato^3^, Panagiotis Dendrinos^1^, Stephen Gaughran^3^, Panagiotis Kasapidis^4^, Alexander Kopatz^5^ & Astrid Vik Stronen^2,6^

^1^ MOm/Hellenic Society for the Study and Protection of the Monk seal, Solomou 18, 10682 Athens, Greece.

^2^ Department of Biology, Biotechnical Faculty, University of Ljubljana, Večna pot 111, 1000 Ljubljana, Slovenia.

^3^ Sackler Institute for Comparative Genomics, American Museum of Natural History, Central Park West at 79^th^ Street, New York, NY 10024, USA.

^4^ Institute of Marine Biology, Biotechnology and Aquaculture, Hellenic Centre for Marine Research, Gournes Pediados, P.O. Box 2214, 71003 Irakleion, Crete, Greece.

^5^ Norwegian Institute for Nature Research (NINA), 7485 Trondheim, Norway.

^6^ Department of Biotechnology and Life Sciences, Insubria University, via J. H. Dunant 3, 21100 Varese, Italy.

^7^ These authors contributed equally: Alexandros A. Karamanlidis and Tomaz Skrbinšek.

* email: [akaramanlidis@gmail.com](mailto:akaramanlidis@gmail.com)

**Methods**

**Molecular analysis**

Genomic DNA was extracted using the Qiagen DNeasy® Blood & Tissue extraction kit, following the manufacturer’s protocol. Extracted DNA was diluted 1:1 in water. Primers were selected from prior success in studies with Mediterranean (*Monachus monachus*) and Hawaiian monk seals (*Neomonachus schauinslandi*). Primer multiplexes were designed and optimized to minimize overlap of PCR product length, with different colored fluorescent dyes used when product lengths potentially overlapped. Multiplex PCRs were prepared using the Qiagen Multiplex PCR Kit, with a recipe of 7.5µL Qiagen Multiplex Master Mix, 0.3µL bovine serum albumin, 1µL template DNA, a volume of fluorescently tagged oligo primers determined by the specific multiplex (Table S1), and a volume of water to bring the total reaction volume to 15µL.

**Table S1.** Primer names, sequence, color, size and quantity used in each of the six multiplex reactions used to study Mediterranean monk seals in Greece. Primer names are marked with superscripts denoting the original publication for that primer set. Superscripts correspond to: 1) [^1^](#_ENREF_1), 2) [^2^](#_ENREF_2), 3) [^3^](#_ENREF_3), 4) [^4^](#_ENREF_4) 5) [^5^](#_ENREF_5) 6) [^6^](#_ENREF_6) 7) [^7^](#_ENREF_7).

**Multiplex 1 (M1)**

| **Primer** | **Primer sequence** | **Color** | **Size (bp)** | **Quantity (µL)** |
| --- | --- | --- | --- | --- |
| Msc03^6^ | F: TGGTCTTTCTTAAGGCCAAG  R: ATATGGAAGCAGCCCAAGTG | Pet | 110 | 0.15 |
| Msc13^6^ | F: CACCTTTGGCTTCCAGTGTC  R: ATTCGGTGGTGGCTTTTATG | Pet | 185 | 0.25 |
| Msc10^6^ | F: CCTCCATCGCTACCATCTTC  R: TGAACGCAAGTGGATGAGTC | Fam | 140 | 0.2 |
| Msc16^6^ | F: GGATTGTGATACACCCTTTCC  R: CTGCATGGATTGACAAAAGG | Fam | 160 | 0.25 |
| Ms504^6^ | F: ATCAGCTATCAGGGGTAGGG  R: GTCATTCCCTAGTGGTAAAGACTC | Fam | 275 | 0.2 |
| Msc19^6^ | F: GGCTATTGGCCAACTGGTAG  R: TTGGCCTGCTCCAATAAGAC | Vic | 130 | 0.25 |
| Msc09^6^ | F: GCCTGATTTGCCTCTTCTTC  R: GCGTCAGAAAGACACAGGAG | Vic | 200 | 0.2 |
| Msc04^6^ | F: CTTTAGTTTCCGGTGTTCAGTG  R: CTCAGGGTTGTGAGTTCAAGC | Ned | 165 | 0.2 |

**Multiplex 2 (M2)**

| **Primer** | **Primer sequence** | **Color** | **Size (bp)** | **Quantity (µL)** |
| --- | --- | --- | --- | --- |
| Ms265^6^ | F: GACTGGTAATTTACGCCCTACC  R: AAGTGTTGGGTTGAAAATTGG | Vic | 160 | 0.1 |
| Ms647^6^ | F: GAACTCCAAACAGCCATTCC  R: CCTGCTCCTTCTTTCTGATCC | Pet | 120 | 0.1 |
| Pv78^3^ | F: GAGTATACCTCCATACTACAC  R: AGTTGTTCTCCTGACCCAAG | Fam | 150 | 0.1 |
| Hg6.3^1^ | F: CAGGGGACCTGAGTGCTTATG  R: GACCCAGCATCAGAACTCAAG | Fam | 210 | 0.1 |
| Msc05^6^ | F: TGGTCTCAAGTTGGAGGATTG  R: AAGCCATTGAGTGTGATGGAC | Ned | 220 | 0.2 |

**Multiplex 3 (M3)**

| **Primer** | **Primer sequence** | **Color** | **Size (bp)** | **Quantity (µL)** |
| --- | --- | --- | --- | --- |
| Msc01^6^ | F: ATTTTAATTATGGGTTACTTTGAACC  R: TCACCATTTAATGCATATGAGC | Vic | 170 | 0.2 |
| Msc23^6^ | F: GCTTCTCTGTTTCTATCTCAAATAAAT  R: CTCCTTCCTGGCTGCTTATG | Pet | 110 | 0.2 |
| Hg6.1^1^ | F: TGCACCAGAGCCTAAGCAGACTG  R: CCACCAGCCAGTTCACCCAG | Pet | 140 | 0.2 |
| Pv17^2^ | F: TTAACAACTCCATTATCATTTGAGCC  R: CTGGTGTGTTAGTGAGGGTTCTGC | Fam | 145 | 0.1 |
| TBPV2^5^ | F: CTCTCCCATCCTCATATTAA  R: GTACTACCCAATATAGAGAC | Fam | 250 | 0.2 |

**Multiplex 4 (M4)**

| **Primer** | **Primer sequence** | **Color** | **Size (bp)** | **Quantity (µL)** |
| --- | --- | --- | --- | --- |
| Neosch2578^7^ | F: CTTTGATTCTGGTCGGGTGC  R: AAGTCACATTGGACGTTGGG | Fam | 230 | 0.2 |
| Neosch3204^7^ | F: ACCAGGAGTAGTCCAAACAGG  R: CACCCGTGTTACAGAGGAAG | Ned | 185 | 0.1 |
| Neosch10588^7^ | F: GATACCAGTGGCCCAAACTC  R: TCAAGCATTCAACCAAGTGC | Vic | 110 | 0.1 |
| Neosch12980^7^ | F: CTGACACCTTCTCTACCGGC  R: CAGTGCCCACGCATGTTTC | Vic | 220 | 0.1 |
| Neosch14583^7^ | F: TTCCAGCTTCTGGTAGTGGG  R: AGTACAGCCTGACACAGACG | Vic | 170 | 0.1 |

**Multiplex 5 (M5)**

| **Primer** | **Primer sequence** | **Color** | **Size (bp)** | **Quantity (µL)** |
| --- | --- | --- | --- | --- |
| Neosch11181^7^ | F: ACTGGCTTTCCTCACTCCTG  R: CGTGAGAATCAACCCTCTGC | Fam | 225 | 0.2 |
| Neosch10909^7^ | F: GTGCGTGCTCTCTATTTTCCC  R: TGTGCATTCCTGTAGCAGAG | Ned | 120 | 0.1 |
| Neosch8549^7^ | F: AGCCTTGTGATGTCAATCTGC  R: TGCGGACAATTCATCCAAATATTAAC | Vic | 170 | 0.15 |
| Neosch16869^7^ | F: GGGACAATTTCTCTCTCTCCC  R: ATGTTCTTTAGTCTCACTTCACG | Pet | 165 | 0.2 |

**Multiplex 6 (M6)**

| **Primer** | **Primer sequence** | **Color** | **Size (bp)** | **Quantity (µL)** |
| --- | --- | --- | --- | --- |
| Neosch17310^7^ | F: TCAAATGGTGGAGGTGAGGG  R: ACTTTTGGTATGTGCTCTGTTCTC | Vic | 125 | 0.1 |
| Neosch6554^7^ | F: TCATTGGTATTCATGTTCCCCC  R: AGCCCAGGCTTGTTGAAATG | Pet | 175 | 0.2 |
| Neosch8948^7^ | F: GTATGTTGTTGCAAACGGCAG  R: AGTGCCTGTTGTGATGGATG | Ned | 125 | 0.1 |
| Pv11^4^ | F: GTGCTGGTGAATTAGCCCATTATAAG  R: CAGAGTAAGCACCCAAGGAGCAG | Fam | 155 | 0.1 |

Samples were run in sets of samples of similar quality. Known high-quality samples had cycling programs of 35 cycles. Lower quality samples were run for 40 cycles to increase amplification. All multiplex PCRs were run with the following thermocycler program: an initial denaturation at 95˚C for 15:00min, followed by cycles of 94˚C for 30s, 56˚C for 1:30min, and 72˚C for 1:30min, with a final extension step at 60˚C for 30:00min. PCR products were diluted 1:50 or 1:100 in water. 1µL of the dilution was added to 9µL of formamide and Liz500 size standard. All samples were repeated three times for each multiplex. Samples were run on an ABI3730 and electropherograms were visualized through the software package GeneMapper v5.0 (Applied Biosystems). Automated allele calls in GeneMapper were checked by eye.

Four out of the 30 loci analyzed (8549, 17310, Hg6_3 and Ms504) were monomorphic and therefore excluded from the downstream analysis.

**Statistical analyses**

*Population structure*

The specifications applied in running STRUCTURE were the following: We used 100 000 burn-in runs, followed by 1 000 000 Markov Chain Monte Carlo (MCMC) repetitions and evaluated *K* = 1 - 5 possible population clusters. We ran each parameter setting three times. We used the admixture model and allowed allele frequencies to be correlated among populations. We examined genetic population structure with and without the LOCPRIOR setting, which incorporates spatial data such as sampling locations in clustering analyses[^8^](#_ENREF_8). We summarized results with STRUCTURE Harvester ver. 0.6.94[^9^](#_ENREF_9) and CLUMPAK[^10^](#_ENREF_10), which included estimates for *∆K*[^11^](#_ENREF_11). We plotted individual assignments using DISTRUCT ver. 1.1[^12^](#_ENREF_12). As uneven sampling can affect STRUCTURE results[^13^](#_ENREF_13), we performed additional analysis with the same parameters, but with independent allele frequencies and without the LOCPRIOR setting, with equalized sample sizes. We retained all Ionian samples (n = 5) and subsampled the two largest clusters (see Results) by randomly selecting from each group n = 10 individuals with high membership to their sampling cluster in earlier analyses of the entire dataset. We also considered the possibility of investigating the possible presence of temporal population structure, but this was not feasible due to uneven sampling distribution across the timeline of the study.

The specifications of the spatial Principal Component Analysis (sPCA) were the following: To correctly model geographic distances we projected the GPS location data for sample locations (WGS84) into the metric Transverse Mercator projection (Greek grid). We modelled spatial connectivity using the Delaunay triangulation[^14^](#_ENREF_14) and used the sPCA scree plot to visually determine the number of components to be interpreted, and Monte Carlo tests with 10,000 permutations to test for the existence of global and local spatial structure[^15^](#_ENREF_15). We ran the analysis in the R statistical environment[^16^](#_ENREF_16) using the *adegenet* package[^17^](#_ENREF_17).

*Hardy-Weinberg Dynamic Subsampling (HWDS) analysis*

In order to explore the size and direction of local deviations from Hardy-Weinberg proportions in a spatially explicit manner, we carried out a Hardy-Weinberg Dynamic Subsampling analysis (HWDS)[^18^](#_ENREF_18) as follows: We constructed a spatial path through the geographic extent of our data, and starting from one side of the path took a “window” subsample of *N_g_* neighboring genotypes/individuals (moving window – spatial “window” of *N_g_* genotypes along the path). We moved this moving window along the path in 1-genotype increments across all genotypes, calculating *H_o_, H_e_* and the *P*-value of a HWE test for each window subsample. Additionally, we also estimated effective population size (*N_e_*) (described in the main text), and calculated average inbreeding (described in the main text) and average q-value of STRUCTURE assignment to the northern Aegean population cluster for each window subsample. In general, this path can be of an arbitrary shape as long as it follows the spatial distribution of samples and/or landscape characteristics. With discrete populations (i.e., the island model) and no gene flow the expectation would be that the window subsamples of genotypes (i.e., individuals) that includes individuals from a single cluster (i.e., area) would be in HWE (i.e., *Ho* = *He*), while the subsamples that would include individuals from neighboring clusters would show the Wahlund effect (*Ho* < *He*)[^19^](#_ENREF_19). Gene flow between the areas would cause deviations from this model, depending on its timing, direction, and magnitude. While a long-term, high gene flow would cause dissolving of population structure, recent short-term gene flow would cause either Wahlund effect with sampling of direct immigrants in the population (*Ho* < *He*), or the “isolate breaking” effect[^19^](#_ENREF_19), where a recent reproduction of immigrants in the recipient population would cause an excess of observed heterozygosity (*Ho* > *He*). This would persist for several generations until, in absence of new immigration, a new equilibrium would be established[^20^](#_ENREF_20). We used the geographic northwest – southeast axis as the moving window path (Fig. S3, Fig. 1C in the main text) and calculated the parameters for each “window” of width *N_g_* = 30 genotypes. We programmed the analysis in R using functions from the *adegenet* package (Note: The R code for this analysis can be requested from T.S.).

**Results**

*Population structure and dispersal*

The results from STRUCTURE Harvester for L(K) and DeltaK supported the presence of K = 3 population clusters (Fig. S1).

**
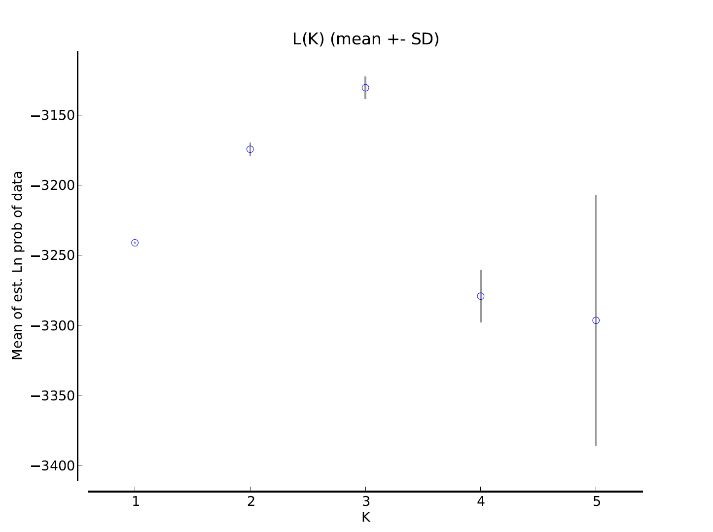
**
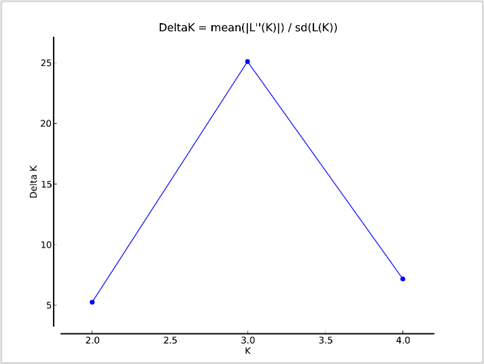


**Figure S1.** L(K) and DeltaK results from STRUCTURE Harvester for K = 1 – 5 population clusters.

The q-values of the animals sampled in the Aegean indicated a clearly-defined, discrete Northern Aegean population and a less clearly-defined Southern Aegean population with admixed animals (Fig. S2).


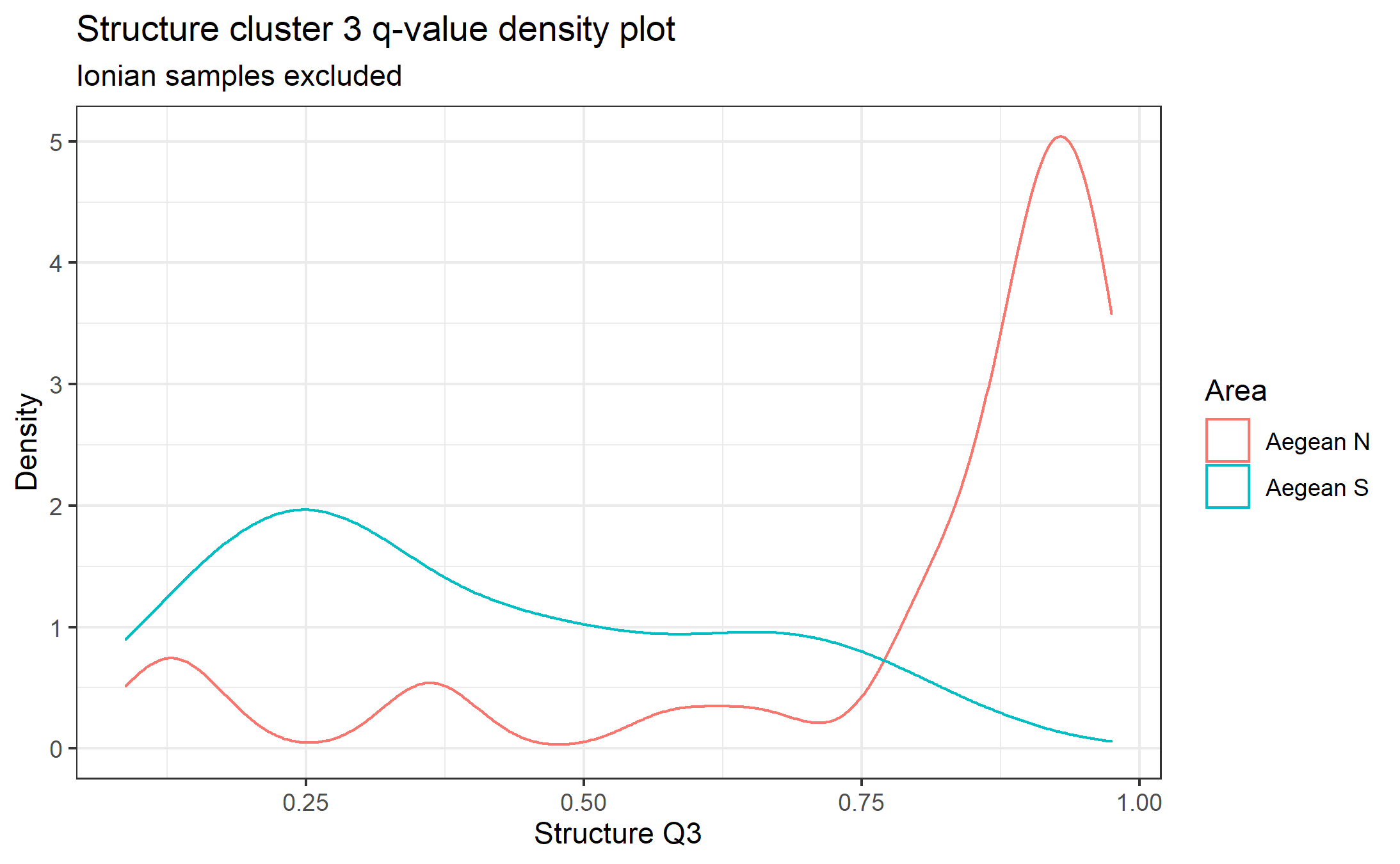


**Figure S2.** Density plot of q-values for Mediterranean monk seals from the northern and southern Aegean Sea.

Of the 47 individuals sampled in the southern Aegean, seven (14.9%) were assigned to the southern Aegean, three (6.4%) to the northern Aegean, and 37 (78.7%) were identified as admixed. Of the 34 individuals sampled in the northern Aegean, 27 (79.4%) were assigned to the northern Aegean, three (8.8%) to the Southern Aegean, and four (11.8%) were found to be admixed. Finally, of the five monk seals sampled in the Ionian, four were assigned to the Ionian, whereas one was assigned to the Northern Aegean. Seventeen individuals were missing > 10% of genotype data across 26 loci, but only five of these were classified as admixed.

Of the 45 females sampled in the study five (11.1%) were classified as migrants, one from the southern to the northern Aegean, three in the opposite direction, and one female sampled in the Ionian Sea was assigned to the northern Aegean Sea. Of the 39 males sampled in the study, two (5.1%) were classified as migrants. Both were individuals sampled in the southern Aegean and assigned to the Northern Aegean. In total, 20 females (44.4%) and 19 males (48.7%) were found to be admixed. Two individuals of unknown sex sampled in the southern Aegean were also identified as admixed. Analyses with equalised sample sizes and without the LOCPRIOR setting also supported the presence of K = 3 population clusters (Fig. S3).


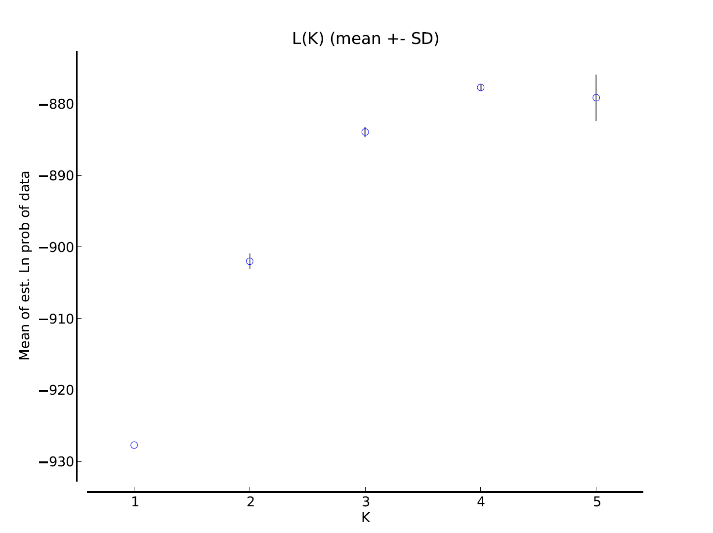

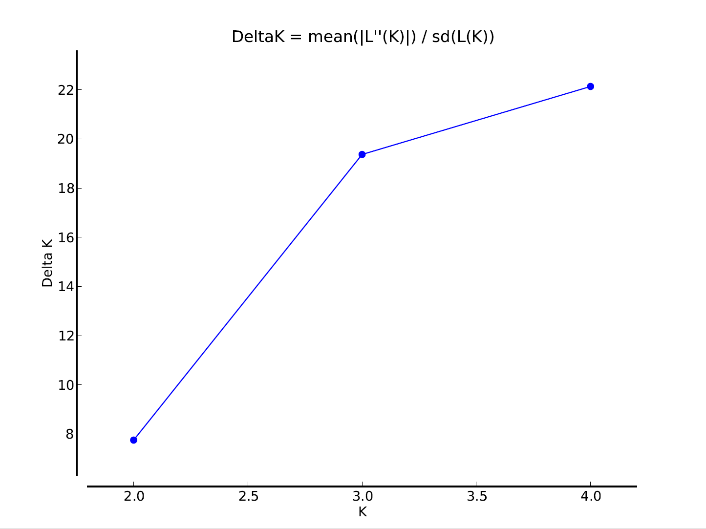


**Figure S3**. L(K) and DeltaK results from STRUCTURE Harvester for K = 1–5 population clusters, with all n = 5 individuals sampled from the Ionian Sea, and n = 10 individuals from the southern and northern Aegean Sea, respectively, randomly selected among animals with high assignment to their sampling cluster in earlier analyses of the entire dataset.

**
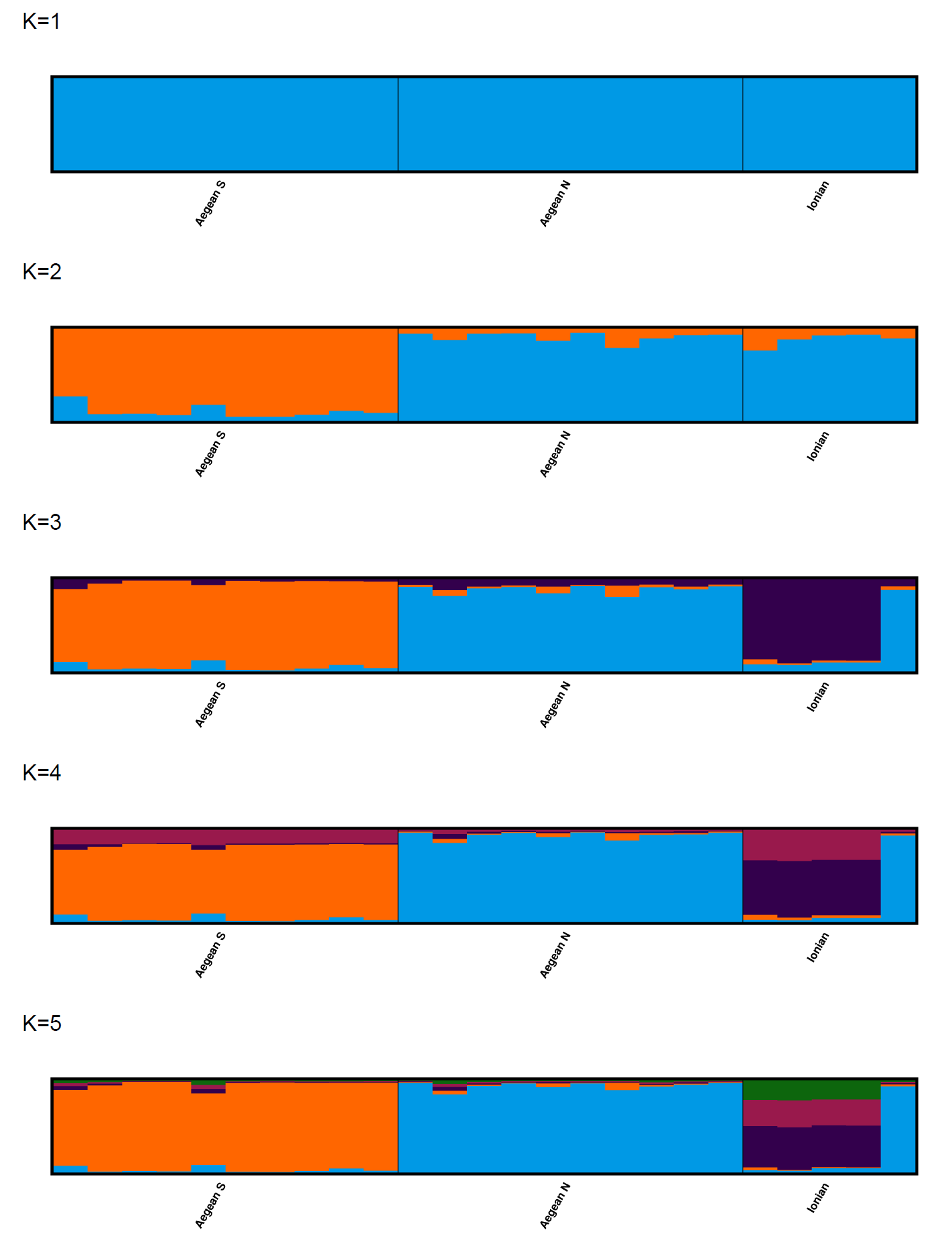
**

**Figure S4.** CLUMPAK results for equalised sample sizes and K = 1-5 population clusters.


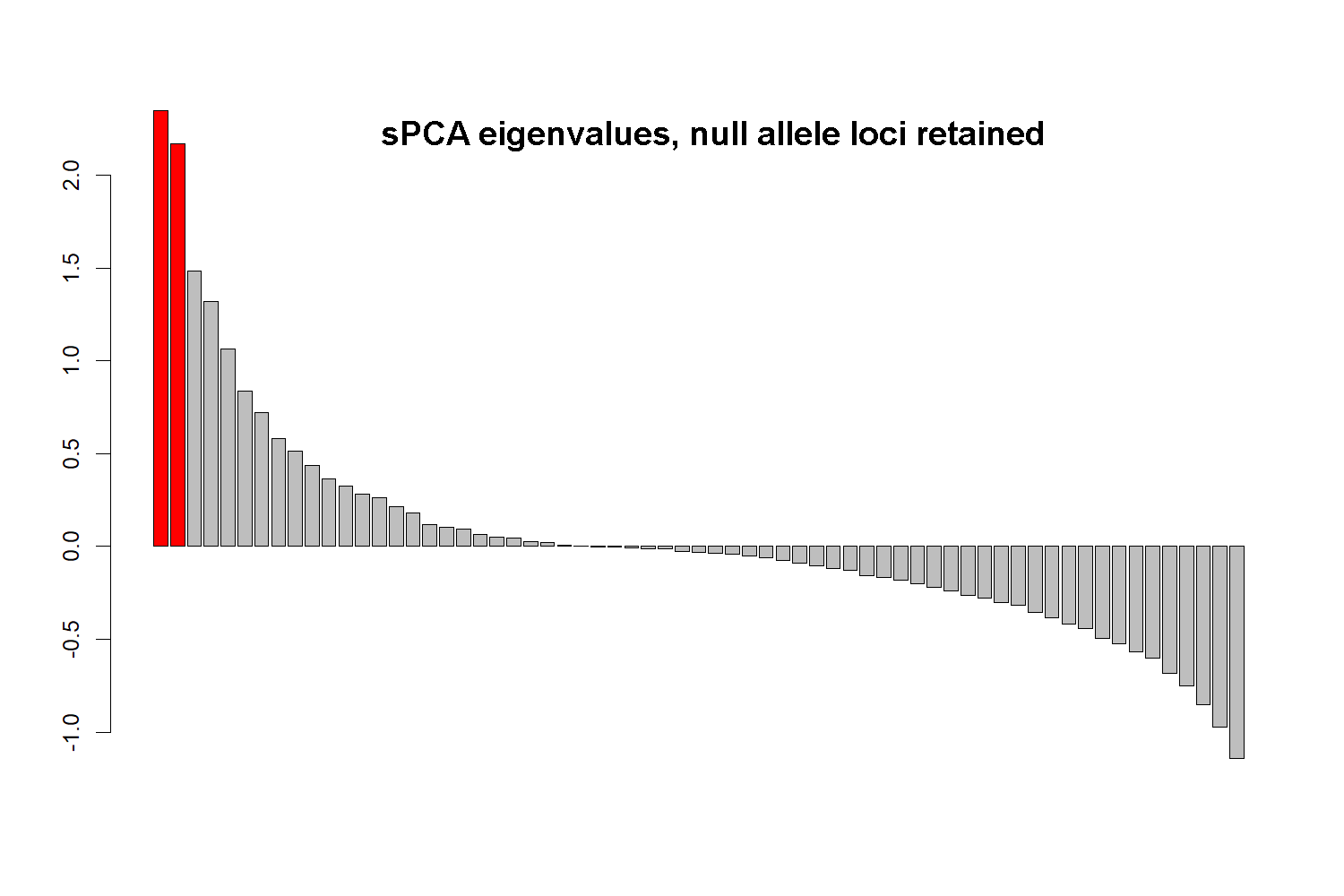


**Figure S5.** sPCA Eigenvalue bar plot for Mediterranean monk seal samples from Greece. The first two global components are informative.


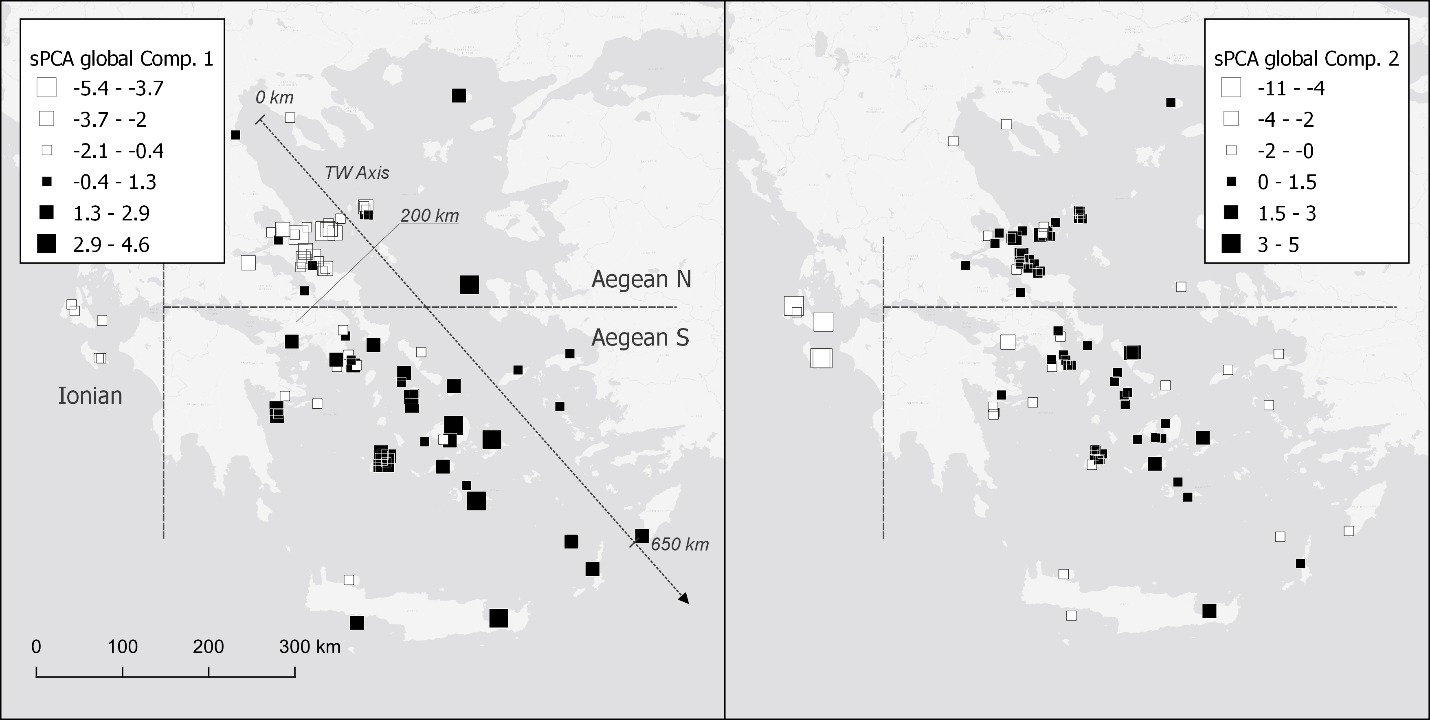


**Figure S6.** Maps of sPCA results for Mediterranean monk seals from Greece. Each square is the geo-location of an individual monk seal, size/color indicate the value. Left: sPCA results for global Component 1; Right: sPCA results for global Component 2 (Figure S6 has been created using QGIS, Version 3.16.0., <https://qgis.org/>).


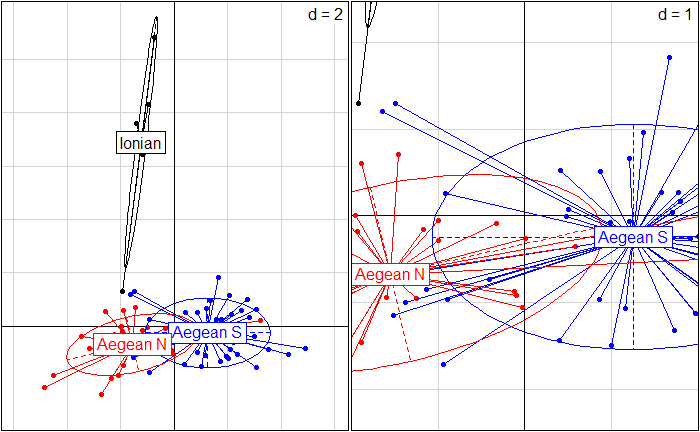


**Figure S7.** sPCA, non-spatial plot of global Components 1 and 2 with marked areas indicating where an individual was sampled. The plot on the right is a close-up of the left plot.

*Individual inbreeding*

COANCESTRY simulations indicated that the TrioML method provided the lowest bias and the highest precision in the individual inbreeding estimates (Table S2). There was little bias in the estimates, but the precision of individual estimates was relatively low (Fig. S4), indicating that the results were useful to evaluate the overall population inbreeding, but less useful to evaluate inbreeding at the individual level. The variance in the estimated individual inbreeding seems high, and there are differences in distribution of inbreeding between northern and southern Aegean (Fig. S5).

**Table S2.** Results of inbreeding simulations performed in COANCESTRY.

| *N* = 4000 | **Ritland** | **LynchRd** | **TrioML** | **DyadML** | **True Value** |
| --- | --- | --- | --- | --- | --- |
| Mean | 0.519 | 0.509 | 0.513 | 0.548 | 0.525 |
| Variance | 0.412 | 0.120 | 0.098 | 0.091 | 0.083 |
| MSE | 0.333 | 0.038 | 0.020 | 0.020 |  |
|  |  |  |  |  |  |
| **Correlation Coefficient** | **Ritland** | **LynchRd** | **TrioML** | **DyadML** | **True Value** |
| Ritland | 1 |  |  |  |  |
| LynchRd | 0.613 | 1 |  |  |  |
| TrioML | 0.480 | 0.918 | 1 |  |  |
| DyadML | 0.483 | 0.920 | 0.997 | 1 |  |
| TrueValue | 0.439 | 0.828 | 0.892 | 0.890 | 1 |


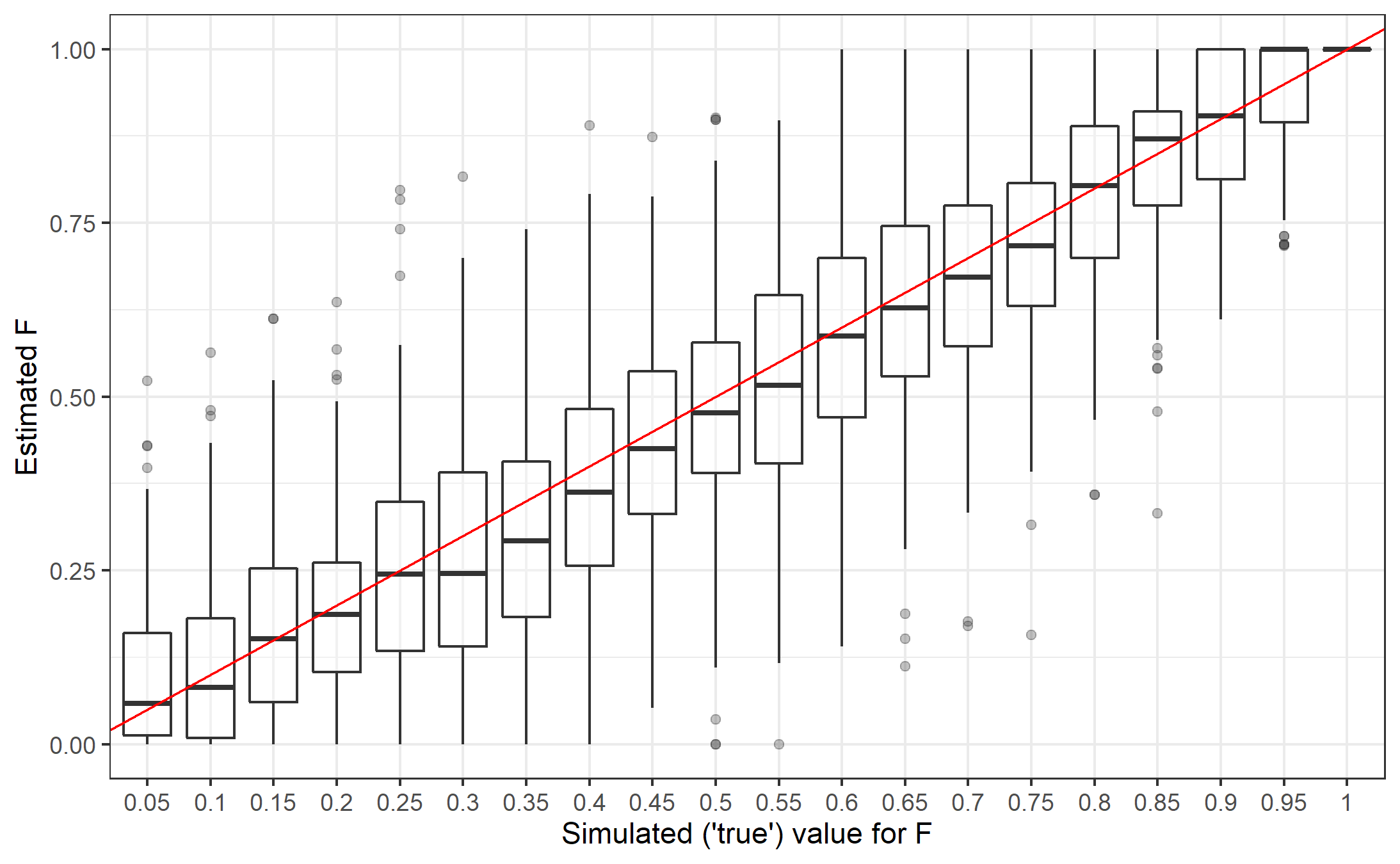


**Figure S8.** Results of inbreeding simulations to estimate the inbreeding coefficient (F) from the simulated data (y-axis), using the TrioML method. Genotypes were simulated at different levels of inbreeding (x-axis), using the markers and allele frequencies observed in this study. The red line shows the “error-free” estimation for each simulated F value.


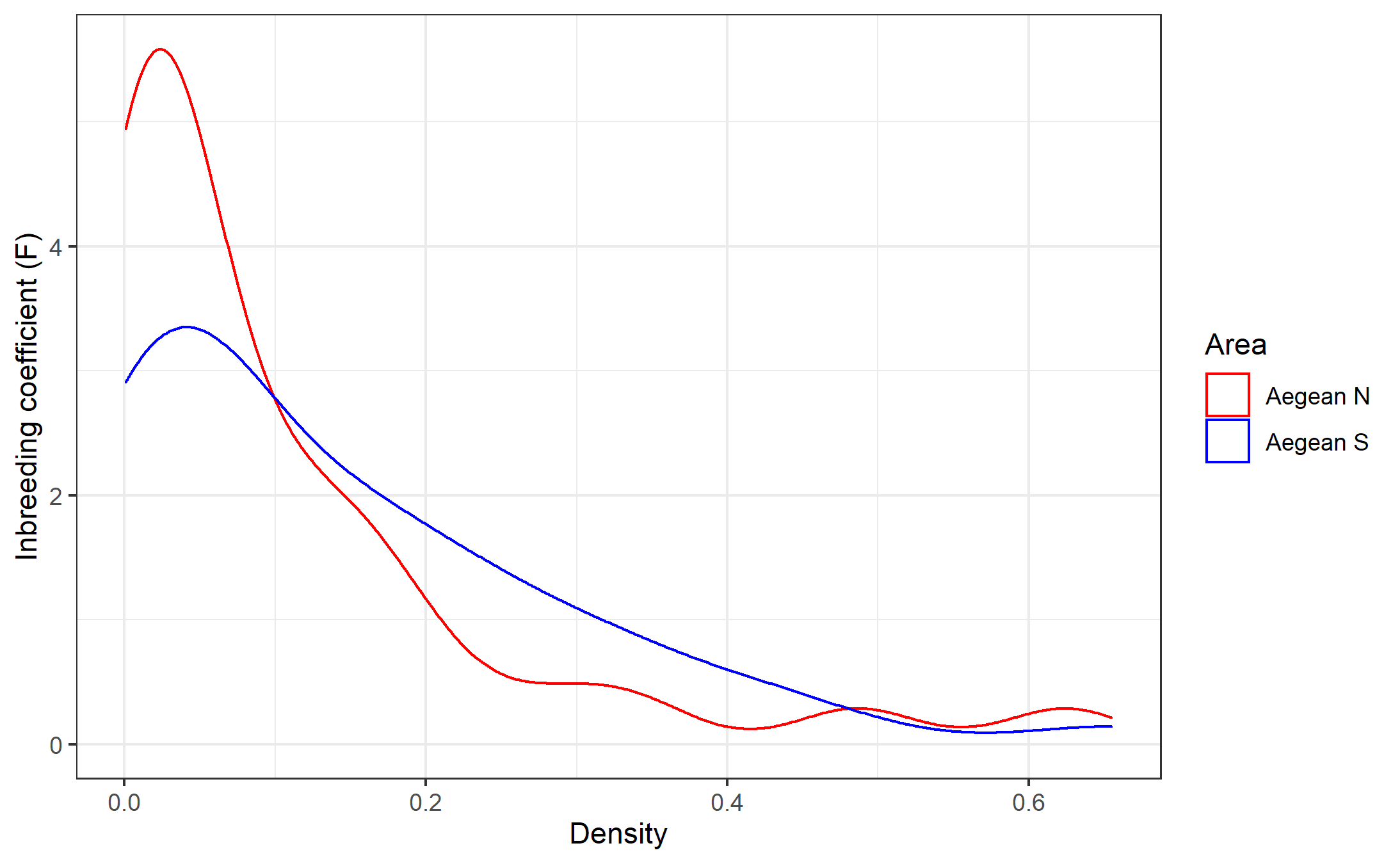


**Figure S9.** Density plot of individual inbreeding coefficients for Mediterranean monk seals in Greece, by area. The TrioML method was used to estimate individual inbreeding. The animals from the Ionian area were excluded due to the small sample size.

**DISCUSSION**

**Effective population size**

Effective population size is a critical parameter for biodiversity conservation, as it describes both, the sensitivity of a population to genetic stochasticity, as well as its evolutionary potential[^21^](#_ENREF_21). However, this parameter is often difficult to assess from real-world data, and results must be interpreted carefully. The linkage disequilibrium method (LD) method assumes no population structure and discrete generations, and both assumptions are severely violated in our analysis. Individuals were sampled over a 22-year period, meaning that they include several generations of animals. [^22^](#_ENREF_22)discuss a reasonable conjecture that if the number of cohorts represented in a sample is approximately equal to the generation length, the LD *N_e_* estimate should roughly correspond to *N_e_* in a generation, which was later supported by[^23^](#_ENREF_23). [^24^](#_ENREF_24)also showed that mixed-age adult samples produce generation *N_e_* estimates, however our samples include a longer time period meaning that several cohorts of parents were included. Because of genetic drift, this causes a temporal genetic structure in the samples and a two-locus Wahlund effect (mixture LD), which results in a downward bias of the LD estimates of *N_e_*[^24^](#_ENREF_24). This means that most of our estimates are probably biased low. However, since the time period is still relatively short (approximately 2 monk seal generations), the bias should not be overly large, particularly in the north Aegean population cluster, where we did not see much deviation from HWE (Fig. 4B).

A similar problem with mixture LD and downward bias in *N_e_* estimates occurs when we have spatial population structure and Wahlund’s effect[^25^](#_ENREF_25). Again, this seems less of a problem in the north Aegean population cluster which seems to be less admixed. It is probably also less of a problem in the northern part of the southern Aegean, where HWDS analysis did not indicate much deviation from HWE, probably because most animals were admixed and there were not many direct immigrants from N or S. However, as we move further to the south, we can expect this downward bias to occur. Fig. 3D (main text) nicely shows these phenomena. TW samples in the Northern Aegean population cluster provided *N_e_* estimates that were close to the estimate obtained using all samples from that area (28.5; 19.3 - 47.3 CI). The estimate increases when animals become more admixed, but then continues to decrease as the TW moves southward and Wahlund’s effect becomes more prominent (Fig. 3B).

Despite all mentioned problems with estimating *N_e_* we can safely say that the effective population size of the monk seal population in the Aegean is low. We were able to obtain the most reliable estimate for the northern Aegean population cluster, and despite the possible low bias of the estimate the effective population size most likely doesn’t meet the rule-of-the-thumb minimum viable population size criteria of *N_e_ >* 50 that would allow the population to avoid inbreeding. Mixing of both Aegean population clusters does increase estimates of effective population size, and despite the southern Aegean population cluster not providing much unique genetic diversity, just the increase in the total pool of breeders seems to be enough to slow down both, the genetic drift and the increase of inbreeding, enough to make the entire Aegean population marginally viable. However, these higher estimates of *Ne* may be driven by the dissolution of linkage in the first generations of admixing, and the long-term effect may be less dramatic.

**References**

1. Allen, P. J., Amos, W., Pomeroy, P. P. & Twiss, S. D. Microsatellite variation in grey seals (*Halichoerus grypus*) shows evidence of genetic differentiation between two British breeding colonies. *Mol. Ecol.* **4**, 653-662 (1995).

2. Goodman, S. J. *Molecular population genetics of the European harbour seal (Phoca vitulina) with reference to the 1988 phocine distemper virus epizootic* PhD thesis thesis, University of Cambridge, (1995).

3. Coltman, D. W., Bowen, W. D. & Wright, J. M. PCR primers for harbour seal (*Phoca vitulina concolour*) microsatellites amplify polymorphic loci in other pinniped species. *Mol. Ecol.* **5**, 161-163 (1996).

4. Goodman, S. J. Patterns of extensive genetic differentiation and variation among European harbor seals (*Phoca vitulina vitulina*) revealed using microsatellite DNA polymorphisms. *Mol. Biol. Evol.* **15**, 104-118 (1998).

5. Pastor, T. *et al.* Low genetic variability in the highly endangered Mediterranean monk seal. *J. Hered.* **95**, 291-300 (2004).

6. Schultz, J. K., Marshall, A. J. & Pfunder, M. Genome-wide loss of diversity in the critically endangered Hawaiian monk seal. *Diversity* **2**, 863-880. (2010).

7. Mihnovets, A. N. *et al.* A novel microsatellite multiplex assay for the endangered Hawaiian monk seal (*Neomonachus schauinslandi*). *Con. Gen. Res.* **8**, 91-95 (2016).

8. Pritchard, J. K., Wen, X. H. & Falush, D. *Documentation for STRUCTURE software: Version 2.3*, <<http://www.ccg.unam.mx/~vinuesa/tlem09/docs/structure_doc.pdf>> (2010).

9. Earl, D. A. & vonHoldt, B. M. STRUCTURE HARVESTER: a website and program for visualizing STRUCTURE output and implementing the Evanno method. *Con. Gen. Res.* **4**, 359-361 (2012).

10. Kopelman, N. M. *et al.* CLUMPAK: a program for identifying clustering modes and packaging population structure inferences across K. *Mol. Ecol. Res.* **15**, 1179-1191 (2015).

11. Evanno, G., Regnaut, S. & Goudet, J. Detecting the number of clusters of individuals using the software STRUCTURE: a simulation study. *Mol. Ecol.* **14**, 2611-2620 (2005).

12. Rosenberg, N. A. DISTRUCT: a program for the graphical display of population structure. *Mol. Ecol. Notes* **4**, 137-138 (2004).

13. Toyama, K. S., Crochet, P.-A. & Leblois, R. Sampling schemes and drift can bias admixture proportions inferred by STRUCTURE. *Mol. Ecol. Res.* **00**, 1-17 (2020).

14. Upton, G. & Fingleton, B. *Spatial data analysis by example. Volume 1: Point pattern and quantitative data*. (John Wiley & Sons Ltd., 1985).

15. Jombart, T., Devillard, S. & Durfour, A.-B. Revealing cryptic spatial patterns in genetic variability by a new multivariate method. *Heredity* **101**, 92-103 (2008).

16. R: A language and environment for statistical computing (R Foundation for Statistical Computing, Vienna, Austria, 2020).

17. Jombart, T. adegenet: a R package for the multivariate analysis of genetic markers. *Bioinformatics* **24**, 1403-1405 (2008).

18. Karamanlidis, A. A. *et al.* History-driven population structure and assymetric gene flow in a recovering large carnivore at the rear-edge of its European range. *Heredity* **120**, 168-182 (2018).

19. Wahlund, S. Zusammensetzung von Populationen und Korrelationserscheinungen vom Standpunkt der Vererbungslehre aus betrachtet. *Hereditas* **11**, 65–106 (1928).

20. Cornuet, J. M. & Luikart, G. L. Description and power analysis of two tests for detecting recent population bottlenecks from allele frequency data. *Genetics* **144**, 2001-2014 (1996).

21. Waples, R. S. in *Population viability analysis* (eds S.R. Beissinger & D.R. McCullough) 147-168 (University of Chicago Press, 2002).

22. Waples, R. S. & Do, C. H. I. Linkage disequilibrium estimates of contemporary Ne using highly variable genetic markers: a largely untapped resource for applied conservation and evolution. *Evol. Appl.* **3**, 244-262 (2010).

23. Robinson, J. D. & Moyer, G. R. Linkage disequilibrium and effective population size when generations overlap. *Evol. Appl.* **6**, 290-302 (2013).

24. Waples, R. S., Antao, T. & Luikart, G. Effects of overlapping generations on linkage disequilibrium estimates of effective population size. *Genetics* **197**, 769–780 (2014).

25. Neel, M. C. *et al.* Estimation of effective population size in continuously distributed populations: there goes the neighborhood. *Heredity* **111**, 189-199 (2013).
